# Non-cystic macular thickening on optical coherence tomography as an alternative to fluorescein angiography for predicting retinal vascular leakage in early stages of uveitis

**DOI:** 10.1038/s41598-022-17701-2

**Published:** 2022-08-05

**Authors:** Nazanin Ebrahimiadib, Zahra Kianzad, Mohammad Zarei, Samaneh Davoudi, Hamid Riazi-Esfahani, Fatemeh Bazvand, Zahra Mahdizad, Bobeck S. Modjtahedi

**Affiliations:** 1 Ophthalmology Department, Tehran University of Medical Sciences, Tehran, Iran; 2 Ocular Immunology and Uveitis Foundation, Waltham, MA USA; 3 Department of Ophthalmology, Boston University School of Medicine, Boston, MA USA; 4 Department of Ophthalmology, Southern California Permanent Medical Group, Baldwin Park, CA USA; 5 Eye Monitoring Center, Kaiser Permanent Southern California, Baldwin Park, CA USA; 6 Department of Research and Evaluation, Southern California Permanente Medical Group, Pasadena, CA USA

**Keywords:** Medical research, Inflammation

## Abstract

To evaluate the relationship between non-cystic thickening of the macula on optical coherence tomography (OCT) and retinal vascular leakage on fluorescein angiogram (FA) in patients with uveitis. A cross-sectional study of patients seen in the uveitis clinic. Patients with any degree of inflammatory cells in the anterior vitreous were included, provided that no macular cyst or subretinal fluid or macular atrophy was observed in OCT. The correlation between OCT features and best corrected visual acuity (BCVA), the degree of inflammation, and FA findings were examined. The severity of vascular leakage in FA was graded for optic nerve, macula and posterior and peripheral leakage. We used generalized estimation equation to assess the associations between macular thickness and volume with angiographic scores. A total of 43 patients (100 exam data) met inclusion criteria. There was a significant relationship between OCT parameters (central macular thickness, 3 mm and 6 mm perifoveal macular thickness as well as total and central macular volume) with angiographic scores (macular, optic disc, posterior and peripheral vascular leakage score) (all P values < 0.0001). The correlation between the 6 mm perifoveal thickness and peripheral vascular leakage score (R = 0.76; P < 0.001) was stronger than the correlation of CMT with this angiographic score (R = 0.69; P < 0.001). Non-cystic thickening of the macula on OCT, especially in perifoveal area, is a reliable predictor of the presence of retinal vascular leakage in patients with uveitis.

## Introduction

Uveitis is an important cause of vision loss and requires complex monitoring of disease activity which relies on both physical exam and multi-modal imaging^1,2^. Early treatment of intraocular inflammation, and associated sequelae, is critical to preserving vision. The degree of uveitis activity has been correlated to macular and retinal nerve fiber layer thickness on optical coherence tomography (OCT)^1,3^. Perivascular retinal thickness on OCT is correlated with uveitis activity and has been suggested as a surrogate for the severity of large vessel leakage on fluorescein angiography (FA)^3,4^.

Retinal vasculitis can be difficult to diagnose on clinical exam and is an important cause of visual morbidity in uveitis patients. It has been reported that one out of 8 uveitis patients has retinal vasculitis, with most not having corresponding signs of systemic vasculitis^5,6^. The Standardization of Uveitis Nomenclature (SUN) Working Group has defined retinal vasculitis as the presence of ocular inflammation and retinal vascular changes including perivascular sheathing and vascular leakage or perivascular leakage with occlusion on FA^7^.

FA is an essential tool in the diagnosis, grading, and monitoring of retinal vascular leakage ^6^ with ultra-wide-field FA allowing for earlier detection and treatment of this entity, potentially resulting in better outcomes^8^. Although FA provides valuable insights into disease activity, it is time consuming, requires fluorescein sodium (which may not always be available in all areas), and carries the risk of adverse events.

Immunomodulatory therapy is often adjusted based on the severity of vascular leakage on FA; however, frequently repeating FAs magnifies the aforementioned challenges with this modality. Identifying surrogate biomarkers on OCT for angiographic leakage may help identify which patients require FA for further assessment of retinal disease activity and also lessen the demand on FA for the assessment and monitoring of retinal vascular leakage.

To the best of our knowledge, no prior studies have evaluated the correlation of macular thickness on OCT with vascular leakage on FA in uveitis patients. This present study sought to characterize OCT features that correlate with the presence, severity, extension and location of retinal vascular leakage on FA.

## Methods

Patients with active uveitis who presented to the Farabi Eye Hospital uveitis service between January and October 2019 were included in this cross-sectional study. We included patients with any sign of inflammation in the anterior vitreous, in the absence of cystoid macular edema or atrophy using OCT. Using slit lamp examination, patients with active inflammation, with the presence of cells in anterior vitreous were eligible for inclusion. Patients with foveal atrophy, intra-retinal cysts/sub-retinal fluid, or epiretinal membrane on macular OCT were excluded as were those with glaucoma, diabetes, cataract surgery within the past six months, and media opacities that precluded retinal assessment. Eyes that had reduced vision from causes not directly related to active inflammation, such as cataract or amblyopia, were not included in analyses exploring visual acuity correlations.

Institutional review board approval was attained from the Tehran University of Medical Sciences and the protocol adhered to the Declaration of Helsinki. Written informed consent was obtained from all patients. Spectral-domain OCT (Heidelberg Engineering Inc, Heidelberg, Germany, software version 6.5.2.0), high speed 6 mm scan, and central 55° FA with seven standard fields were performed for all patients. During the first minute following the intravenous injection of sodium fluorescein, early frames were captured followed by later frames at about at least 4–5 min later. Images of posterior pole and peripheral sweeps were captured for both eyes.

Patients were imaged either at their presentation to the uveitis clinic or during their follow up when they were receiving immunomodulatory therapy. Follow up sessions were included as separate sets of data if they were at least three months apart.

Baseline patient characteristics including age, sex, and history of systemic disease were recorded as were clinical exam features including the presence, location, and degree of intraocular inflammation. Comprehensive ophthalmologic examinations were performed for all patients. Best-corrected visual acuity (BCVA) in LogMAR was assessed.

Macular thickness maps using Early Treatment Diabetic Retinopathy (ETDRS) were assessed in three zones; in the central 1 mm (CMT), between the 1 and 3 mm rings, and between the 3 and 6 mm rings. The central 1 mm and total macular volume were also considered. The thickness values of each nine sector of ETDRS on the topographic map were recorded. The mean thickness of the 4 sectors between the 1 and 3 mm rings was defined as perifoveal 3 mm thickness and the mean thickness of 4 sectors between 3 and 6 mm rings was defined as perifoveal 6 mm thickness. Normative thickness maps and quantitative values for retinal thickness were used, with orange or red coloration in a sector being considered evidence of thickening. OCTs were reviewed to ensure that the segmentation lines were arranged correctly. In case of segmentation error, the image was excluded from the analysis.

Anterior chamber (AC) and anterior vitreous (AV) cells were graded by one uveitis expert (NE), blinded to the result of FA and OCT findings, on a level of 0 to 4+ based on observation of cells in a dilated pupil during slit lamp examination^9^. The size of the slit lamp beam used for evaluation of AC cells was 1 × 1 mm and for AV cells was 1 × 0.5 mm. For the purpose of analysis, the least number of cells observed in the vitreous was assigned a numeric value of + 0.25.

**Figure 1:**
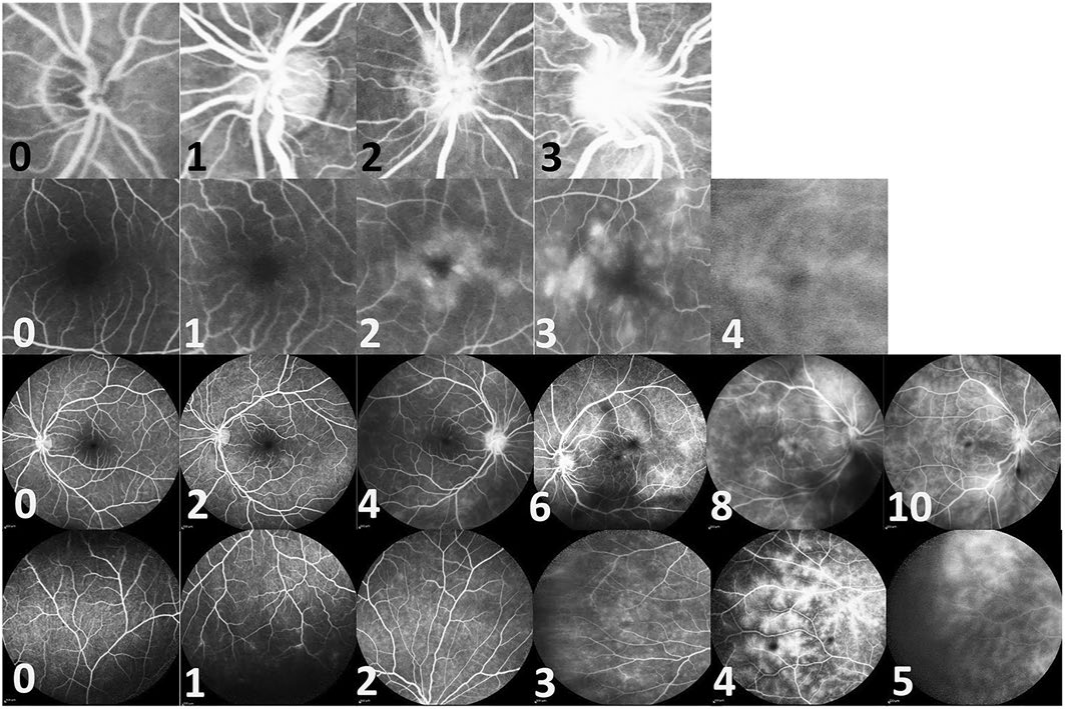
Examples of FA scoring system for grading of inflammatory activity. The numbers below each figure represent the scores. The first row shows the optic disc Hyperfluorescence grading, 0–3 (from left to the right; 0: normal, 1: partial staining of the disc, 2: diffuse leakage without blurring of the disc margin, 3: diffuse leakage and blurring of the disc margin.) The second row shows macular leakage grading, 0–4 (from left to the right; 0: no perifoveal hyperfluorescence, 1: incomplete ring of leakage, 2: complete (360°) leakage but less than 1 disc diameter (DD) wide, 3: complete (360°) leakage of 1–1.5 DD wide, 4: complete (360°) leakage of more than 1.5 DD wide) The third row shows posterior vascular leakage grading, 0–10 macular hyperfluorescence was not included (from left to the right; 0: none, 2: increased visibility of the smallest capillaries or scattered faint capillary leakage, 4: diffuse mild capillary leakage, 6: more intense diffuse leakage with clear distinction between adjacent vascular domains, 8: greater leakage with blending of adjacent leaking domains into each other in less than half of the area of the posterior view (excluding macular hyperfluorescence), 10: greater leakage with blending of adjacent leaking domains into each other in more than half of the area of the posterior view) The fourth row shows the peripheral vascular leakage grading, 0–5 (from left to the right; 0: none, 1: increased visibility of the smallest capillaries or scattered faint capillary leakage, 2: diffuse mild capillary leakage, 3: more intense diffuse leakage with clear distinction between adjacent vascular domains, 4: greater leakage with blending of adjacent leaking domains into each other in less than half of the area of the peripheral quadrant, 5: greater leakage with blending of adjacent leaking domains into each other in more than half of the area of the quadrant).

A previously defined grading system for FA grading was utilized^10^. This grading system is based on frames peripheral and posterior retinal leakage on frames at least five minutes after infusion of fluorescein. The prototype of this grading is depicted in Fig. 1. Peripheral vascular leakage grading was based on the severity and extent of leakage. The severity of peripheral vascular leakage was graded as 0 (no leakage), 1 (minimal) defined as faint background leakage, 2 (mild) defined as diffuse but mild background leakage, 3 (moderate) defined as more intense leakage without the boundary between vessels being not blended, 4+ (severe) defined as significant leakage which causes blending of leakage areas into each other in less than half of the area of a peripheral quadrant and 5 (very severe) defined as diffuse blending of leakage in more than half of the area of a peripheral quadrant. The scoring was done separately for each quadrant such that a maximum score of 5 can be assigned to each quadrant with an overall maximum score of 20 being possible for all four quadrants when combined. Posterior vascular leakage has three components: retinal vascular leakage posterior to the equator, macular leakage, and optic nerve leakage. Macular and optic nerve leakage were graded separately and were not considered in grading leakage posterior to the equator which was scored as follows: 0 (none), 2 (faint background leakage), 4 (diffuse but mild background leakage), 6 (more intense leakage but the boundary between vessels is not blended), 8 (significant leakage which causes blending of leakage area into each other in less than half of the area), 10 (diffuse blending of leakage in more than half of the area). The grading of macular leakage was based on observation of the ring of leakage and can have a maximum score of 4; 0 (no perifoveal hyperfluorescence), 1 (incomplete ring), 2 (complete ring < 1-disc diameter in size), 3 (complete ring 1–1.5 disc diameter in size), 4 (complete ring > 1.5 disc in size). The grading of optic disc hyperfluorescence can have a maximum score of 3 and includes 0 (normal staining of scleral rim), 1 (partial staining), 2 (diffuse leakage without blurring of the disc margin), and 3 (diffuse leakage with blurring of the disc margin). Scoring was performed by an expert who was blinded to the result of OCT findings and slit lamp examination.

### Statistical analysis

Descriptive statistics were calculated as the mean, median, range, 25th and 75th percentile, and standard deviation. Associations between macular thickness and volume with angiographic scores were estimated using a generalized estimation equation (GEE) approach that accounted for involving neighboring eyes and repeated measures. In addition, the correlation between OCT parameters and cells in the anterior vitreous, anterior chamber, and BCVA were investigated using partial correlation coefficients which also compensates for related observations in bilateral subjects and follow up measurements. Strength of relations during the follow up period was evaluated by interaction analysis within GEE. To compensate for multiple comparisons, Bonferroni’s method was applied. All statistical analyses were performed with the SPSS software (IBM Corp., released 2016; IBM SPSS Statistics for Windows, Version 24.0, Armonk, NY: IBM Corp.). P values ≤ 0.05 were considered significant.

## Results

A total of 100 exam data of 43 patients met inclusion criteria. Forty-one patients had bilateral retinal vascular leakage on FA. Eight of 41 patients with bilateral uveitis/retinal vascular leakage had a history of systemic disease: tuberculosis (2 patients), Behcet’s disease (2 patients), HLA-B27+ ankylosing spondylitis (2 patients), Juvenile idiopathic arthritis (one patient) and psoriasis (one patient). The follow up data of 8 patients were also included; two eyes of one patient at one year follow up, 6 eyes of 3 patients at the 6th month follow up time and 8 eyes of 4 patients at the 3rd month. Baseline characteristics are summarized in Table 1. Three eyes with significant cataract, one eye with substantial posterior synechiae, and two eyes with amblyopia were excluded from vision analyses. Three patients (4 eyes) had a history of cataract surgery greater than six months before study enrollment.

**Table 1:** Demographic and clinical characteristics of the study population.

Number of patients (eyes, data)	43 (87)
**Age**	
Years ± SD (range)	21 ± 14 (6–64)
**Gender**	
Male	24
Female	19
**Lens status**	
Pseudophakic	2 patients (2 eyes)
Aphakic	1 patient (2 eyes)
**Laterality of uveitis**	
Unilateral	2
Bilateral	41
**Type of uveitis (patients)**	
Intermediate	12
Posterior	31
**Grading of cells**	
AC median (range, 25th percentile, 75th percentile)	0 (0–1, 0, 0)
AV median (range, 25th percentile, 75th percentile)	0 (50–3, 0.25, 1)
Best corrected visual acuity decimal (range)	9/10 (5/10–10/10)
LogMAR; mean (SD)	0.1 (0.2)
**Diagnosis**	
Undefined	35 patients
TB	2
JIA	1
Behcet’s disease	2
Psoriasis	1
AS	2

*AC* anterior chamber, *AV* anterior vitreous, *BCVA* best corrected visual acuity, *LogMAR* logarithm of the minimum angle of resolution, *TB* tuberculosis, *JIA* Juvenile Idiopathic Arthritis, *AS* ankylosing spondylitis.

The mean CMT was 297.25 ± 34.44 μm (min = 227 and max = 397). Mean perifoveal 3 mm thickness was 371.55 ± 25.60 μm and mean 6 mm perifoveal thickness was 333.49 ± 23.81 μm. The mean total macular volume was 9.63 ± 0.65 mm^3^ and the mean central macular volume was 0.23 ± 0.02 mm^3^. The median peripheral and posterior vascular leakage scores were 8 (range: 0–19, 25th percentile = 4, 75th percentile = 9) and 2 (range: 0–10, 25th percentile = 2, 75th percentile = 5), respectively. The median macular and optic disc leakage scores were 1 (range: 0–4, 25th percentile = 0, 75th percentile = 3) and 1 (range: 0–3, 25th percentile = 1, 75th percentile = 1), respectively. Table 2 demonstrates the correlations between OCT measures and FA, clinical exam, and BCVA.

**Table 2:** The correlation (p values and correlation coefficients) of macular parameters using OCT with angiographic and examination findings.

Inflammation indices/macular indices on OCT (mean ± SD)	Angiographic findings				Examination findings		
	Macular leakage score	Optic disc leakage score	Posterior vascular leakage score	Peripheral vascular leakage score	AC inflammation	AV inflammation	BCVA (0.10LogMAR)
CMT (297.25 ± 34.44 μm)	< 0.001 R = 0.40	0.001 R = 0.36	< 0.001 R = 0.63	< 0.001 R = 0.69	0.35	0.26	0.74
Perifoveal 3 mm thickness (371.55 ± 25.60 μm)	< 0.001 R = 0.40	0.001 R = 0.34	< 0.001 R = 0.61	< 0.001 R = 0.65	0.62	0.68	0.41
Perifoveal 6 mm thickness (333.49 ± 23.81 μm)	< 0.001 R = 0.49	< 0.001 R = 0.49	< 0.001 R = 0.71	< 0.001 R = 0.76	0.87	0.34	0.65
Central macular volume (0.23 ± 0.02 mm^3^)	< 0.001 R = 0.38	0.002 R = 0.33	< 0.001 R = 0.61	< 0.001 R = 0.67	0.39	0.34	0.80
Total macular volume (9.63 ± 0.65 mm^3^)	< 0.001 R = 0.48	< 0.001 R = 0.46	< 0.001 R = 0.70	< 0.001 R = 0.75	0.92	0.28	0.97

R = Correlation Coefficient.

**Figure 2:**
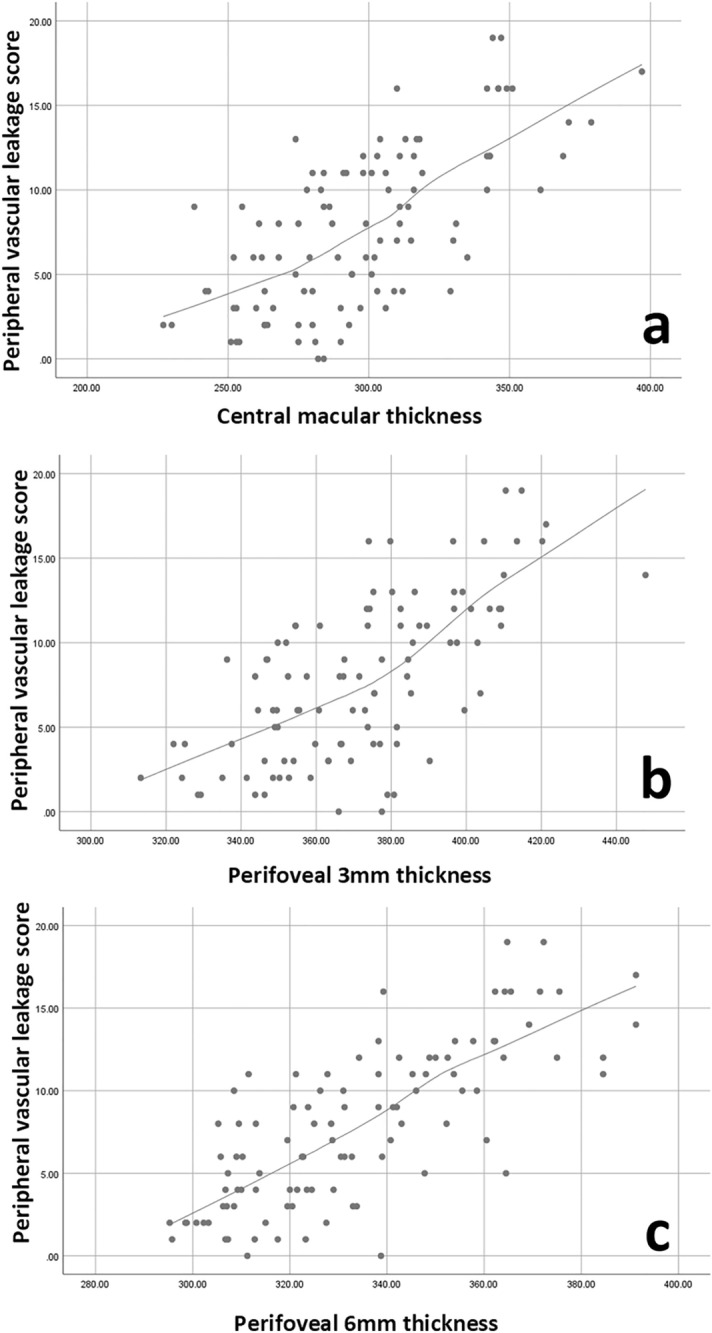
Scatterplot showing the correlation between peripheral vascular leakage score and CMT (**a**) as well as perifoveal 3 mm (**b**) and 6 mm (**c**) thickness.

There was a significant correlation between CMT and peripheral vascular leakage on FA (Fig. 2). CMT additionally correlated with the macular, optic disc and posterior leakage scores. There were statistically significant correlations between 3 and 6 mm perifoveal thickness and angiographic peripheral vascular leakage (Fig. 2).

**Figure 3:**
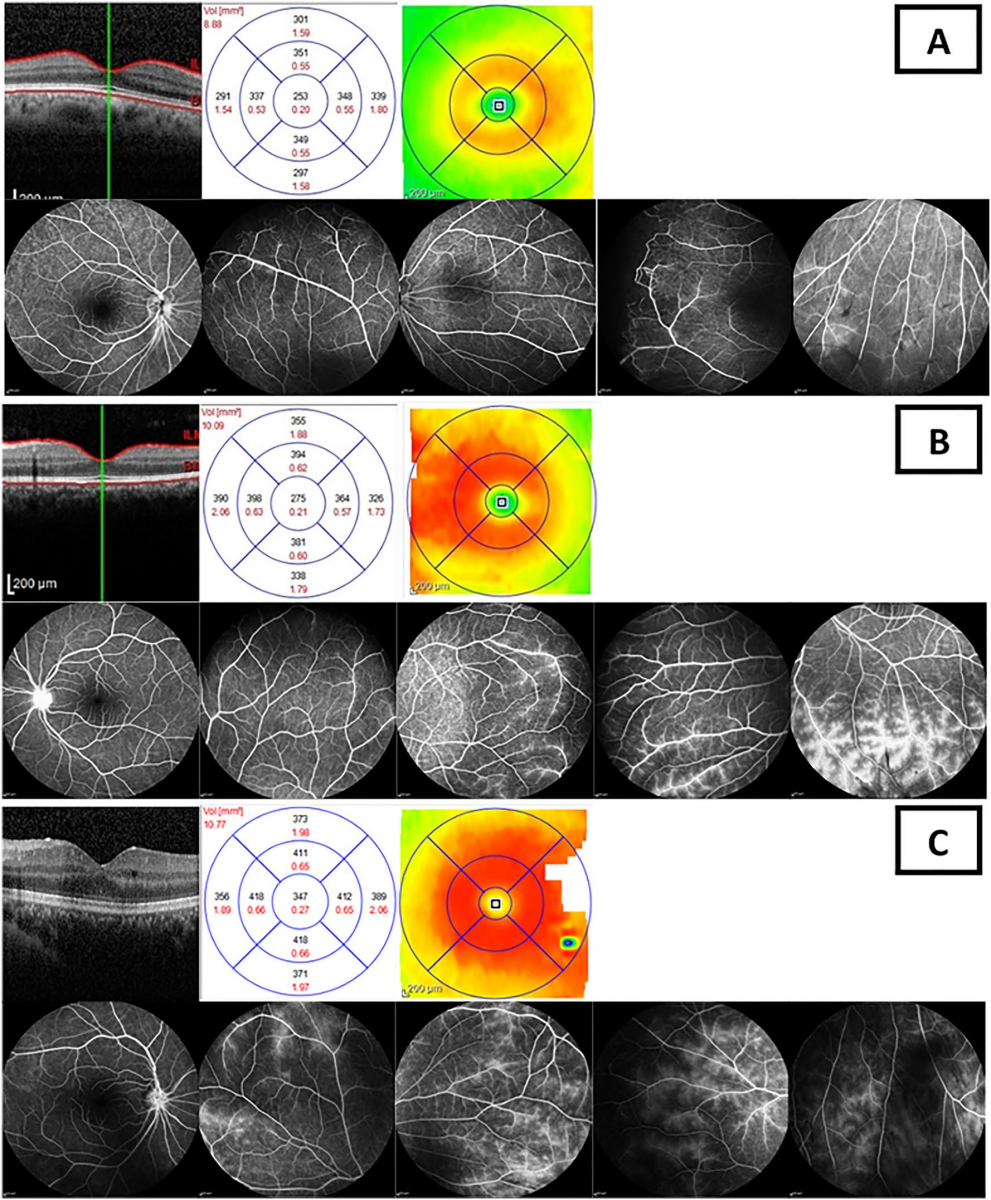
Concomitant OCT and fluorescein angiography (FA) images of three patients with various levels of inflammation to demonstrate the correlation between macular thickness and angiographic score. (**A**) The prototype of concomitant OCT and FA with mild severity of leakage; top row illustrate the OCT images: from left to the right macular B scan centered at the fovea, average thickness and retinal thickness color map (CMT = 259 μm, Mean 3 mm perifoveal thickness = 348.5 μm, mean 6 mm perifoveal thickness = 309 μm, total macular volume = 8.94 mm^3^ and central macular volume = 0.20 mm^3^). The lower row shows FA images from posterior pole and peripheral retina demonstrating optic disc, macular and posterior leakage scores of 0 and total peripheral leakage score of 4. (**B**) The prototype of concomitant OCT and FA with moderate severity of leakage; the top row illustrates the OCT findings: from left to the right macular B scan centered at the fovea, average thickness and retinal thickness color map (CMT = 275 μm, Mean 3 mm perifoveal thickness = 384.25 μm, mean 6 mm perifoveal thickness = 352.25, total macular volume = 10.09 mm^3^ and central macular volume = 0.21 mm^3^). The lower row shows FA images from posterior pole and peripheral retina demonstrating optic disc and posterior leakage score of 2, macular leakage score of 0, and total peripheral leakage score of 8. (**C**) The prototype of concomitant OCT and FA with high grade severity of leakage; the top row illustrates the OCT findings: from left to the right macular B scan centered at the fovea, average thickness and retinal thickness color map (CMT = 347 μm, Mean 3 mm perifoveal thickness = 414.75 μm, mean 6 mm perifoveal thickness = 372.25 μm, total macular volume = 10.77 mm^3^ and central macular volume = 0.27 mm^3^). The second row shows FA images from posterior pole and peripheral retina demonstrating optic disc and posterior leakage scores of 0, macular leakage score of 2 and total peripheral leakage score of 16.

There was also a significant relationship between central and total macular volume with peripheral as well as posterior leakage scores. Regression analysis demonstrated that every unit increase peripheral vascular leakage score was associated with an average increase of 5.8 and 132.6 mm^3^ in the total and central macular volume, respectively. Figure 3 represents the correlation of concomitant OCT and FA, in three different levels of inflammatory activity.

A subgroup analysis was done for patients with peripheral vascular leakage who did not have leakage in the posterior retina. In this group of patients, the peripheral vascular leakage score was significantly correlated with BCVA, AC inflammation, and AV inflammation (p = 0.002, p = 0.028 and p = 0.003, respectively) Regression analysis showed that after adjusting for angiographic macular and posterior leakage, the mean CMT, 3 mm perifoveal thickness, and 6 mm perifoveal thickness were still significantly correlated with peripheral leakage score (p = 0.02, p = 0.02 and p = 0.05, respectively).

Reviewing the normative thickness map revealed that in the area between the 1- and 3-mm circle, an average of 3.5 sectors had a red or orange color indicative of thickening; and in the area between 3- and 6-mm circle, an average of one sector with these colors was present. In the area between 1- and 3-mm circle, 96 (96%) eyes had at least one sector of red or orange color and 63 (63%) eyes had all four sectors with these colors. In the area between 3- and 6-mm circle, only three (3%) eyes had all sectors in red or orange and 43 (43%) eyes were free of any sectors with red or color coding.

Comparing the R values (based on the interaction analysis within GEE) between different OCT parameters and angiographic scores demonstrated that the correlation between the 6 mm perifoveal thickness and peripheral vascular leakage score (R = 0.76; P < 0.001) was stronger than the correlation of CMT with this angiographic score (R = 0.69; P < 0.001)^11^, although both p values were significant.

## Discussion

The management of uveitis patients relies on in-depth history taking, physical exam, and imaging. The findings presented herein demonstrate that non-cystic or spongiform macular thickening is strongly correlated with the presence, severity, and extent of retinal vascular leakage on FA. Increases in the volume of Muller cells are the first histopathologic findings in macular edema and correspond to spongiform macular edema on OCT. When cellular dysfunction occurs, fluid first accumulates inside retinal cells resulting in tissue swelling without cystic changes. As swelling progresses, cystic changes eventually form within the macula^12^. The goal of the present study was to characterize OCT changes before they progressed to more obvious and severe signs of uveitic damage such as cystic macular edema or subretinal fluid. Two previous studies found that OCT findings could serve as a noninvasive measure of inflammatory activity in birdshot retinochoroiditis due to the correlation between perivascular thickening on OCT and retinal vascular leakage on FA^3,4^. Unlike the present investigation, these prior studies did not describe the severity, extent and location of retinal vascular leakage on FA and did not investigate the correlation between such parameters and OCT findings.

FA gives valuable information regarding the presence, extent and severity of retinal vascular leakage; however, there are no widely accepted standardized grading systems for uveitis leakage. This study relied on a previously published grading system that is used in the authors’ daily practice^10^. Vascular leakage involves increasing areas of peripheral retinal and can grow into the posterior pole as inflammation progresses. Additionally, the intensity of leakage both in the periphery and posterior pole is a sign of uveitis severity. These results indicate that when the fovea contour is preserved, the level of retinal thickening in the fovea and perifoveal area on OCT can reliably predict angiographic leakage. To validate the importance of peripheral vascular leakage in causing macular thickening, we performed a subgroup analysis on those patients with peripheral vascular leakage who were lacking vascular leakage in posterior pole (including the maula). Analysis showed that peripheral vascular leakage in the absence of posterior pole involvement is significantly correlated thickening on OCT. Therefore, identifying even small areas of thickening on the OCT may help with monitoring disease activity and could reduce the frequency of FA ordering. Patients in this study had lower grades of AC inflammation than AV inflammation, possibly because the focus of this study was those with posterior segment inflammation and additionally because topical corticosteroids can suppress AC inflammation more than AV inflammation. In clinical practice it can be difficult to determine if AV cells are from intermediate uveitis or spill over cells from the AC. Macular changes on OCT can help guide which patients may have posterior uveitis.

Patients in this study had thickening on OCT with preserved foveal contours and without cystoid macular edema. As such, they were in relatively earlier or milder stages of inflammation. Visual acuity and AV inflammation were not correlated with macular OCT parameters. This may be because CMT that predicts visual acuity was near normal in our patients. Additionally, the amount of AV cells in this cohort was small with a mean score of 0.5. All angiographic scores were significantly correlated with foveal and perifoveal thickness and volume, with further distance from the fovea having a strong correlation with inflammatory indicators. Examining color changes on the OCT thickness MAP can also provide insights into the presence of peripheral vascular leakage with the doughnut between the 1- and 3-mm circle most likely to show areas of thickening in those with peripheral vascular leakage.

This study demonstrates that non-cystic thickening on macular OCT, especially in the perifoveal area, strongly corresponds to retinal vascular leakage. Color maps on OCT can help highlight macular thickening and indicate the presence of retinal vascular leakage. A significant correlation was seen between peripheral vascular leakage and OCT changes, even in the absence of posterior vascular leakage on FA. This may be from inflammatory mediators which induce intra-cellular inflammation. One of the limitations of the current study is lack of lack of a standard scoring system for the inflammatory findings in FA. Naturally, the angiographic information is subjective and qualitative, however, with using our angiographic scoring, we tried to objectify these findings. Another limitation is interobserver evaluation in scoring FA, however, in another study from our center, this scoring system was shown to have a high interclass correlation coefficient of 0.986^10^. Larger and prospective studies are necessary to support this correlation and determine which OCT features correspond to changes in retinal vascular leakage over time, especially with regards to treatment response. In addition, including uveitis patients with cystic macular edema in another study would be useful to evaluate the validity of our findings in this subset of patients.

## Data Availability

The datasets used and/or analyzed during the current study are available from the corresponding author on reasonable request.
